# Recycled Multi-Material Packaging Reinforced with Flax Fibres: Thermal and Mechanical Behaviour

**DOI:** 10.3390/polym14204423

**Published:** 2022-10-19

**Authors:** Irene Bavasso, Claudia Sergi, Teodoro Valente, Jacopo Tirillò, Fabrizio Sarasini

**Affiliations:** Department of Chemical Engineering Materials Environment and UdR INSTM, Sapienza-Università di Roma, Via Eudossiana 18, 00184 Roma, Italy

**Keywords:** polymer matrix composites, multi-material packaging, flax fibres, mechanical properties, thermal properties, polymers and aluminium (PolyAl), recycling

## Abstract

In this work, the use of a recycled mix stemming from the treatment of multilayer aseptic packaging used in the food and beverage industry is proposed as the matrix for short fibre composites reinforced with flax fibres, to generate value-added materials in contrast to the more common end-of-life scenario including energy recovery. This is expected to be a preferred choice in the waste hierarchy at the European level. A commercially available material (EcoAllene) obtained from multilayer packaging recycling was compounded with short flax fibres up to 30 wt.% by twin screw extrusion, with a view to enhancing its poor mechanical profile and broadening its applications. Composites were in depth analyzed by thermogravimetric analysis and differential scanning calorimetry, which highlighted the complex nature of this recycled product, a limited nucleation ability of flax fibres and a lower thermal stability due to the premature degradation of natural hemicellulose and cellulose, though featuring in any case onset degradation temperatures higher than 300 °C. Composites’ mechanical properties were assessed in tension, bending and impact conditions, with remarkable improvements over the neat matrix in terms of stiffness and strength. In particular, at 30 wt.% fibre content and with 5 wt.% of maleated coupling agent, an increase in tensile and flexural strength values by 92% and 138% was achieved, respectively, without compromising the impact strength. The effectiveness of flax fibres confirmed by dynamo-mechanical analysis is beneficial to the exploitation of these composites in automotive interiors and outdoor decking applications.

## 1. Introduction

Plastic packaging represents a key component in the food industry, providing improved food storage, handling, transportation and preservation [1]. Despite these positive attributes, it is also seen as the source of a significant amount of waste because of the worldwide exploitation of fossil-based polymers [2]. In Europe, for instance, between 2009 and 2019, the volume of plastic packaging waste generated per inhabitant increased by 24%.

The biggest issue in using plastic packaging is the post-consumer waste, and one way to face this situation is the design and development of new packaging materials based on biopolymers characterized by a lower environmental impact due to the exploitation of renewable resources and/or due to improved end-of-life management options such as composting or anaerobic digestion [3]. Despite the global market of biopolymers being expected to grow over the next several years, with global production capacities estimated to reach 7.59 million tonnes by 2026 [4], thus achieving a share in global plastic production higher than two percent, this is not enough in the short period. In fact, the replacement of petroleum-based plastics with bio-based biodegradable ones is still hindered by their higher cost and some technical issues, such as the narrow processing window, lower toughness and mechanical properties [5].

When dealing with waste management methods, according to the waste hierarchy of the European Directive on waste management [6], the third best option after prevention and preparing for re-use, is mechanical recycling; therefore, the life cycle of every plastic material can be considered sustainable only if its disposal options include recycling. As regards plastic packaging materials, in 2019, approximately 41% of plastic packaging waste was recycled in the European Union, but this trend needs to be significantly improved to keep pace with increasing plastic production. Currently, PET (polyethylene terephthalate) and HDPE (high-density polyethylene) bottles show the best outlook in terms of high-quality recycled materials, due to well-established selective collection, sorting and efficient recycling processes, leading to high-quality recycled materials [7]. In this regard, additional problems can be envisaged when multilayer polymer-coated paperboards are considered, i.e., beverage cartons, which generally exhibit complex architectures depending on the specific type of food/beverage to preserve. The widely diffused Tetra Pak^®^ aseptic package consists of a structure made of six layers featuring different contents of paperboard (75 wt.%), LDPE (low density polyethylene, 20 wt.%) and thin (~6.5 µm, 5 wt.%) aluminium foil. Every layer performs a specific role but at the same time poses challenges when it comes to the development of recycling strategies. Tetra Pak is currently striving to reduce the carbon footprint of the packages while making the post-consumer cartons more attractive for recyclers. For instance, the replacement of the aluminium layer, which contributes to a third of the green-house gas emissions linked to base materials used by Tetra Pak, with a fibre-based barrier layer might increase the paper content, thus making the cartons more attractive for paper mills.

These innovative attempts are also coupled with some recycling approaches that have been developed over the years [8]. The most industrially viable process includes at first the removal of the cellulosic part by hydrapulping in paper mills, which is destined to be used for corrugated cardboard or tissue paper, and then the processing of the left overs, which are roughly a mixture of polyethylene and aluminium, known as PolyAl or PE-AL, with the presence of other impurities. The mechanical process involves injection moulding and extrusion steps for a direct transformation of PolyAl material in components for end-market applications. This approach has been investigated in other studies with some success [9,10], while a value-added product under the trade name EcoAllene has been also developed [11]. Also in this case, the resulting polymer pellets can be melt compounded and injection moulded, but the mechanical properties are more appropriate for non-structural or cosmetic applications.

To broaden the industrial applications of these recycled products, Cravero and Frache [12] recently optimized the flammability and combustion behaviour of EcoAllene by developing formulations based on magnesium hydroxide, while other researchers manufactured composite materials based on natural fibres, such as fique [13] and waste flour (rice husk and beech sawdust) [14]. Results showed benefits in terms of tensile and flexural stiffness, but at the expense of strength performance because of poor interfacial adhesion. EcoAllene is a commercially available product which can rely on a consistent and uniform supply in contrast to other recycled materials obtained from heterogeneous plastics. Nonetheless, the mechanical and thermal profiles of such material need to be improved if new and profitable fit end markets are meant to be reached. Promising results have been reported in a previous work dealing with the design and development of basalt-based composites [15], where improvements by 107% and 162% for tensile and bending strength, and by 45% for impact strength were obtained, respectively, at a content of 30 wt.% basalt fibres.

Currently, no studies are available on the manufacturing, mechanical and thermal characterization of EcoAllene-based composites reinforced with flax fibres, which represent the most industrially and scientifically mature plant fiber for the reinforcement of polymer matrices [16,17,18]. Properties and specific features of flax fibres as composites’ reinforcement have been summarized in excellent and in-depth reviews [19,20,21,22]. Flexural, tensile and Charpy impact properties have been supportupported by differential scanning calorimetry, thermogravimetry and dynamic mechanical analysis to highlight the effect of flax fibres and maleated coupling agent on the behaviour of the resulting composites aiming at outlining new industrial applications for this sustainable material instead of incinerators and landfills.

## 2. Materials and Methods

### 2.1. Raw Materials

Chopped flax fibres (F) were supplied by Teillage Vandecandelaere (Bourguebus, France) with a nominal length of 2 mm and without any specific sizing. A EcoAllene^®^ AA00 was used as the matrix, which was provided by Ecoplasteam S.p.a. (Alessandria, Italy). As described elsewhere [15], this is a polyethylene and aluminium waste stemming from the treatment of post-consumer packaging waste. To tailor the flax fibre/matrix interfacial adhesion, a commercially available coupling agent (CA) Polybond^®^ 3009 supplied by Addivant Corporation (Danbury, CT, USA) was used. This is a maleic anhydride modified high density polyethylene (MA-g-PE), featuring a maleic anhydride content in the range of 0.8–1.2%.

### 2.2. Composite Processing

The compounding of the composite formulations was conducted in a parallel co-rotating twin screw extruder (Thermo Scientific Process 11, Thermo Fisher Scientific, Waltham, MA, USA) in accordance with the following temperature profile from hopper to die: 160-160-170-180-180-180-175-175 °C. A screw speed of 150 rpm and a feed rate (single screw feeder) of 5 were used. The Process 11 contains a 40:1 L:D ratio barrel with two 11 mm fully segmented, co-rotating screws. A standard screw configuration was employed, with conveying elements (helix pitch of 1 L/D) interspaced among three mixing sections obtaining alternating elements with 0° and 90° (length ¼ L/D). Finally, a discharge extrusion element (1 ½ L/D) with a single lead geometry was used to generate the needed extrusion pressure.

After compounding, the specimens for the thermal and mechanical analyses were obtained by injection moulding (Haake MiniJet II Pro, Thermo Fisher Scientific, Waltham, MA, USA) with a mould and loading cylinder kept at 50 °C and 175 °C, respectively. Samples were injected with a pressure of 600 bar held for 10 s and a post pressure of 60 bar maintained for another 10 s. Then, the mould was manually opened and prepared for the next injection cycle. All the manufactured composite formulations are listed in Table 1.

### 2.3. Mechanical Characterization of Composites

Tensile tests were conducted on a universal testing machine Z010 equipped with a 10 kN lod cell (Zwick/Roell, Ulm, Germany) in displacement control according to ISO 527-2. A cross-head speed of 10 mm/min with type 1BA samples (gauge length = 30 mm) was used, while the strain was measured with a Zwick/Roell contacting extensometer.

Three-point bending (ISO 178) tests were performed on the same equipment with a cross-head speed of 5 mm/min, a support span length of 64 mm and a pre-load of 2 N. Strain was recorded with a displacement transducer in contact with the samples.

Charpy impact tests were carried out as per ISO 179-2 regulation in an edgewise mode by using notched (type A) samples (80 × 10 × 4 mm), a span of 62 mm and a CEAST/Instron 9340 (Pianezza, Italy) instrumented drop weight tower operated at an impact velocity of 2.90 m/s and 13.5 J. For all mechanical tests, the results were reported as the average of at least five replicates.

### 2.4. Thermal Characterization of Composites

Thermogravimetric analysis (TGA) was conducted on 45 mg of composites which were placed in an alumina pan and heated from 25 °C to 800 °C in a nitrogen atmosphere (50 mL/min) with a heating rate of 10 °C/min. A Setsys Evolution system by Setaram (Caluire, France) was used.

As for differential scanning calorimetry (DSC), specimens (~9.8 mg) were placed in a concavus aluminium crucible with a pierced lid and tested in a DSC 214 Polyma by Netzsch GmbH (Selb, Germany). Two heating cycles and one cooling cycle were carried out in a nitrogen atmosphere (60 mL/min) according to the following schedule: first heating from −40 °C to 220 °C (5 min hold), cooling to −40 °C (10 min hold), and second heating to 220 °C; all steps were conducted with a rate of 10 °C/min.

The DMA 242 E Artemis by Netzsch GmbH was used for dynamic mechanical analysis (DMA) in three-point bending. Samples (60 × 10 × 4 mm) were heated at 2 °C/min from −160 °C up to 70 °C under a frequency of 1 Hz and strain amplitude of 50 µm. For all thermal characterizations, the results are the average of three replicates.

### 2.5. Morphological Characterization

Specimens after tensile and Charpy impact tests were sputter coated with gold and imaged by a field-emission scanning electron microscope (FE-SEM) Mira3 by Tescan (Brno, Czech Republic).

## 3. Results

### 3.1. Thermal Characterization of Composites

**Figure 1:**
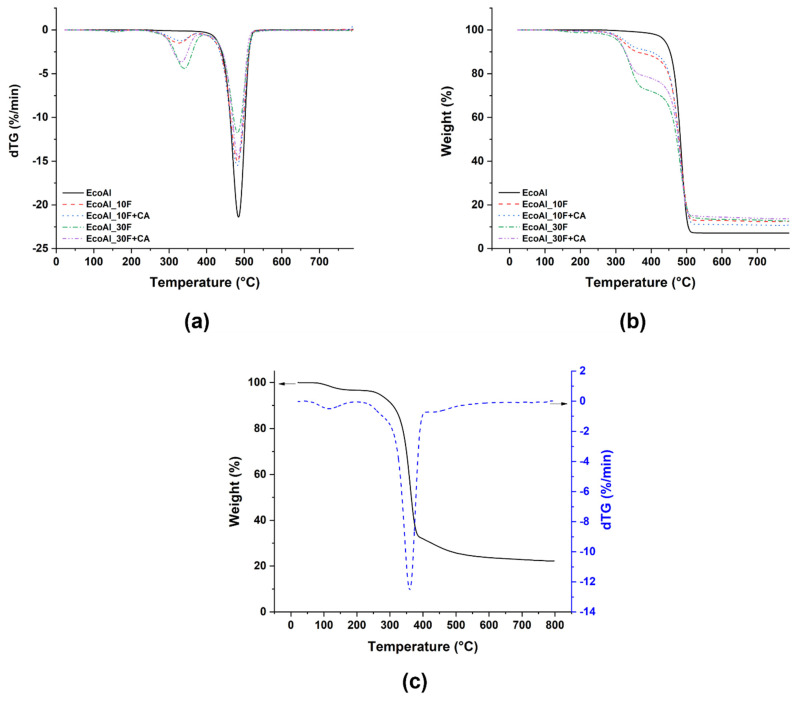
(**a**) Derivative (dTG) and (**b**) TG curves for all EcoAllene-based formulations along with (**c**) neat flax fibres.

Table 2 summarizes the typical temperature values that were used to assess the thermal stability of EcoAllene matrix upon addition of flax fibres, as obtained from representative TGA curves displayed in Figure 1.

T_5%_ and T_10%_ stand for the temperatures related to a weight loss of 5% and 10%, respectively, whereas the temperature corresponding to the maximum degradation rate is indicated with T_max_. As observed in [15], pure EcoAllene featured a degradation behaviour characterized by a single step, with a maximum degradation temperature close to 478 °C, which is ascribed to LDPE and in line with other studies [12,13], where slight deviations can be related to the presence of impurities, EcoAllene being a compound obtained from the recycling of post-consumer aseptic packaging. Another issue to consider is the amount of aluminium in the mix, which is around 7.5 wt.% in the present study, well below the more conventional amounts found in the range 15–25 wt.% [23], which is likely to be ascribed to the patented process by Ecoplasteam specifically focused on reducing the amount of aluminium in the final PolyAl mixture.

As already observed for basalt fibres [15], even with flax fibres, the thermal stability of composite formulations decreased with increasing flax content (Table 2), though the maximum degradation temperature remained unaffected. The change in the global degradation behaviour, marked by the occurrence of a second peak around 340–350 °C (Figure 1), is typical of the degradation of flax fibres and, in particular, of their main constituent, i.e., cellulose [24]. The TG curves of neat flax fibres are included in Figure 1c as a reference. The anticipated onset of thermal degradation in terms of T_5%_ is due to the degradation of non-cellulosic components such as pectin and hemicellulose [25], well visible in Figure 1c [26]. It is worth mentioning the positive role played by the coupling agent, which shifted the onset of thermal degradation at higher temperatures compared to non-compatibilized formulations, as a result of a better interfacial adhesion [27], as confirmed by SEM micrographs shown in Section 3.2. This behaviour confirms that, in this material system, the coupling agent and its amount are appropriate, without causing a dispersion effect, as observed elsewhere [28].

**Figure 2:**
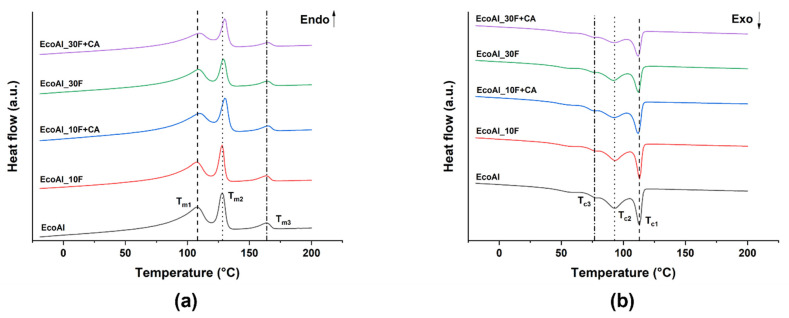
DSC second heating scan (**a**) and cooling scan (**b**) for EcoAllene-based composites.

EcoAllene is not simply a mixture of LDPE and aluminium, as it can be easily inferred from the DSC thermograms shown in Figure 2, displaying the cooling and second heating scans for all EcoAllene-based composite formulations. Relevant thermal parameters are collected in Table 3.

In Figure 2a, three endothermic events can be seen, marked as T_m1_, T_m2_ and T_m3_, which are in line with the melting points of LDPE, HDPE and PP, respectively [29,30]. Three peaks can be recognized also in the cooling curves (Figure 2b). While those labelled as T_c2_ and T_c3_ are due to the crystallization of LDPE with its characteristic broad melting range and low peak shape related to long branches and wide molecular weight distribution [29,31], T_c1_ is instead likely due to HDPE with the crystallization of PP overlapped, which occurs in the range 110–115 °C [32,33]. Flax fibres did not significantly affect the crystallization and melting temperatures, though these temperatures were higher compared to those found in basalt-EcoAllene composites, suggesting an easier crystallization and a more stable crystalline morphology, respectively. The slight nucleating effect of flax fibres on polyolefins, enhanced by the presence of a coupling agent, is in agreement with other studies [34,35].

### 3.2. Mechanical and Dynamo-Mechanical Characterization of Composites

**Figure 3:**
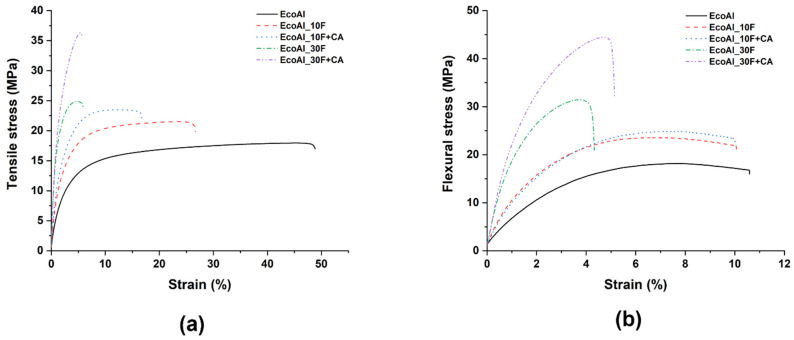
Representative (**a**) tensile and (**b**) three-point bending curves of EcoAllene-based composites.

**Figure 4:**
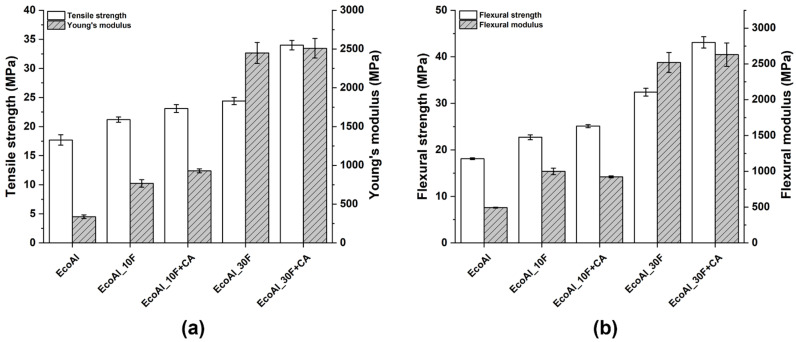
(**a**) Tensile and (**b**) flexural properties of EcoAllene-based composites.

Typical stress vs. strain curves under tensile and bending loading conditions are shown in Figure 3a,b, respectively, while the relevant mechanical properties are collected in Figure 4.

**Figure 5:**
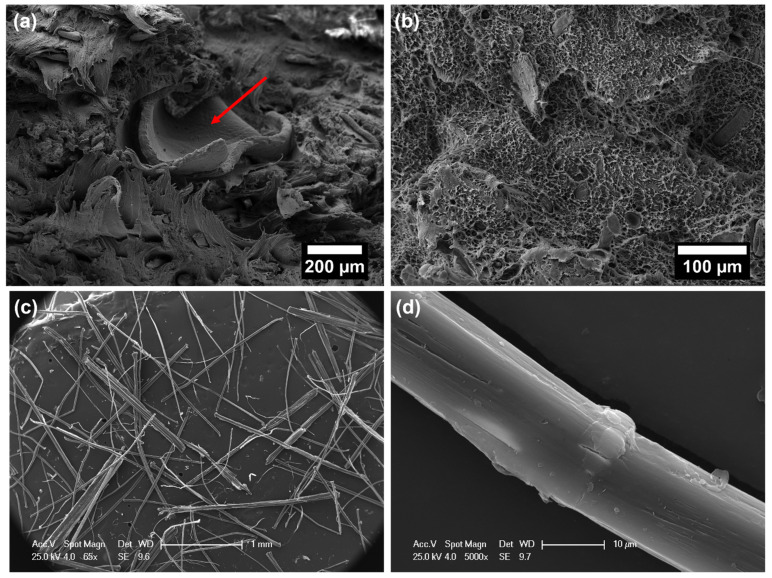
SEM micrographs of neat EcoAllene: (**a**) after tensile test and (**b**) after Charpy impact test; (**c**,**d**) representative SEM micrographs of as-received chopped flax fibres.

**Figure 6:**
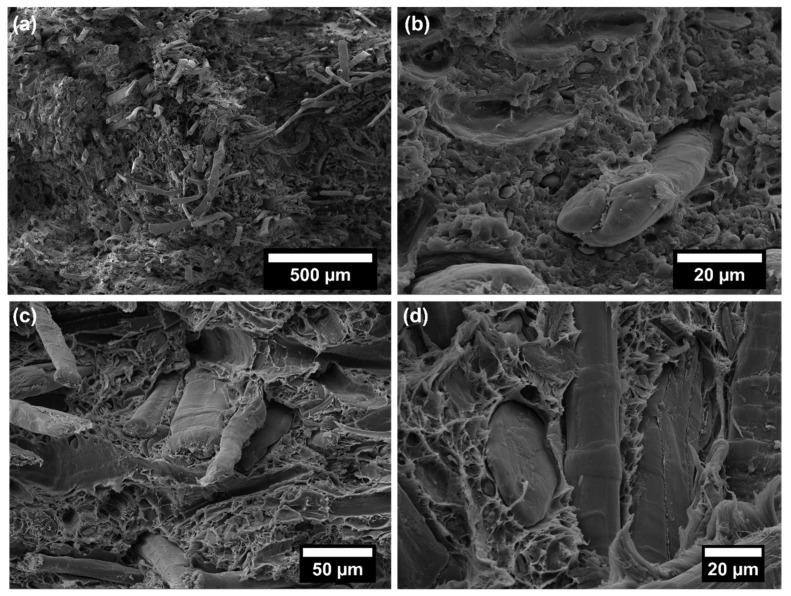
SEM micrographs of EcoAl_30F: (**a**,**b**) after tensile test; (**c**,**d**) after Charpy impact test.

The poor quasi-static mechanical properties of neat EcoAllene, being a mixture based mainly on LDPE and a limited content of aluminium, are significantly enhanced upon the addition of increasing content of flax fibres. In particular, the tensile modulus increased by 127% and 625% as the flax fibre content passed from 10 wt.% to 30 wt.% compared to unfilled EcoAllene, respectively. A similar trend was observed for tensile strength, with improvements over the neat matrix by 20% and 38% at 10 wt.% and 30 wt.% flax fibre content, respectively. While the increased stiffness was expected, the progressive increase in strength was quite unexpected, as Ayrilmis et al. [14] found a decreasing trend in tensile and flexural strength with an increasing amount of sawdust or rice husk flour. This behaviour, reported also for basalt-based composites [15], can be interpreted in terms of an efficient stress transfer at the flax fibre/matrix interface, as supported by SEM micrographs of the fracture surfaces (Figure 5 and Figure 6). For the sake of clarity, in Figure 5c,d, the morphology of as received flax fibres is included, which exhibits the typical morphology with kink bands (Figure 5d) [36]. The ductile behaviour of neat EcoAllene is evident in Figure 5a, where an aluminium flake (indicated by a red arrow) is protruding from the matrix.

**Figure 7:**
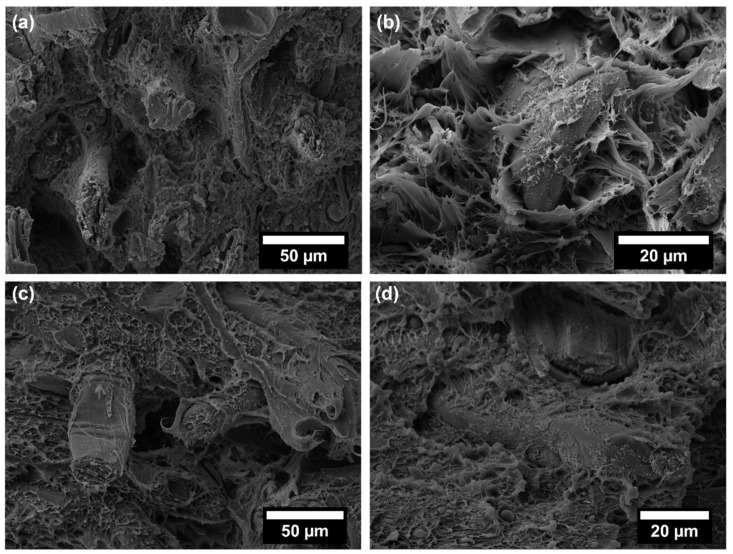
SEM micrographs of EcoAl_30F + CA: (**a**,**b**) after tensile test; (**c**,**d**) after Charpy impact test.

This ductility is reduced by the presence of flax fibres (Figure 6), with evidence of pull-out phenomena and debonding, though matrix residues sticking on flax fibre surface (Figure 6b) are present, thus supporting the existence of a sufficient fibre/matrix interfacial adhesion, likely promoted by impurities (additives) included in the commercial polymer mixture. Coupling agent significantly increased the interfacial adhesion by ester linkages occurring among hydrophilic OH groups and acid anhydride groups from coupling agent [37,38], but without a remarkable reduction in ductility, which was still observed at the micro-scale on the fracture surface (Figure 7). In this case, flax fibres appear well embedded in the matrix, with reduced pull-out and debonding phenomena. Coupling agent enabled a better dispersion of flax fibres inside the matrix, which hindered the formation of fibre clusters which might behave as stress intensifiers. This balanced the increased fibre/matrix interfacial adhesion, enabling a good amount of plastic deformation even at 30 wt.% fibre content, especially effective in bending tests, where the state of stress is complex involving tension, compression and shear. This behaviour is further confirmed by flexural toughness, evaluated as the area under stress–strain curves. Composites exhibited a higher amount of energy absorption per unit volume compared to neat matrix, with moduli of toughness of 2010.4 ± 55.1 and 1671.2 ± 47.3 kJ/m^3^ at 10 wt.% and 30 wt.% flax fibres with coupling agent, respectively. The higher ultimate strength is the key influencing factor for the higher modulus of toughness compared to the neat matrix (1547.6 ± 53.4 kJ/m^3^). Extrinsic toughening due to the presence of fibres with improved fibre/matrix interfacial adhesion and better dispersion is responsible for improved energy absorption because a large amount of strain energy can be stored in the fibres and subsequently dissipated after fracture, instead of being used to propagate cracks. The reduction of strain at break in tension is the limiting factor for tensile toughness, but also, in this case, at 30 wt.% flax fibres, the presence of coupling agent caused an increased modulus of toughness (1520.2 ± 39.7 kJ/m^3^) with respect to formulation without a coupling agent (1325.4 ± 53.8 kJ/m^3^).

**Figure 8:**
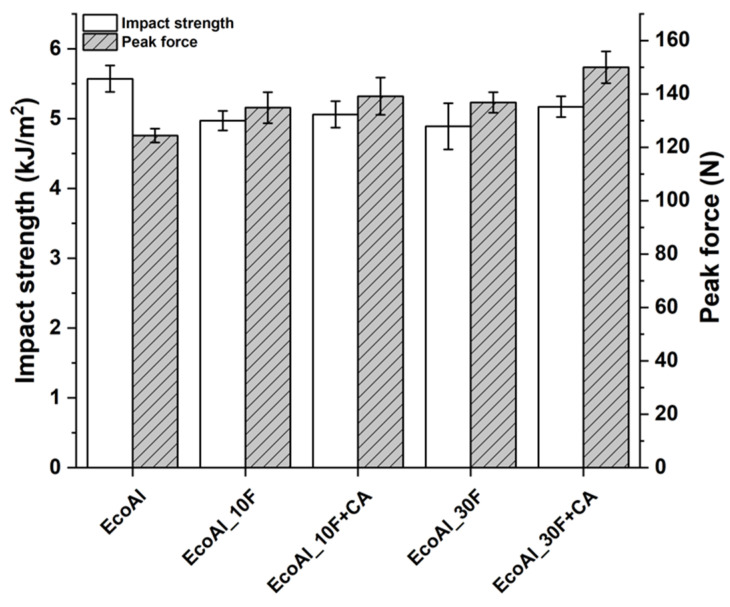
Charpy impact test results of EcoAllene-based composites.

No detrimental effect on Charpy impact strength of EcoAllene matrix was detected, as shown in Figure 8. Fracture surfaces (Figure 5b, Figure 6c,d and Figure 7c,d) featured a limited ductility and, considering composites, higher impact peak forces and impact strengths were achieved in modified formulations. The stronger fibre/matrix adhesion resulted in greater work of fracturing of the interphase that limited crack propagation [39]. This effect was counteracted by the lower fracture resistance of flax fibres compared to synthetic fibres [15], leading to an overall impact strength similar to the untreated matrix.

The positive mechanical results obtained by EcoAllene reinforced with flax fibres were compared in Table 4 against those of virgin polymer matrices reinforced with lignocellulosic fibres. It can be seen that composite formulations manufactured in the present study are in line with those based on virgin HDPE and biopolymers, thus suggesting potential applications (but not limited to) in the fields traditionally covered by wood plastic composites, such as plastic lumber decking boards.

**Figure 9:**
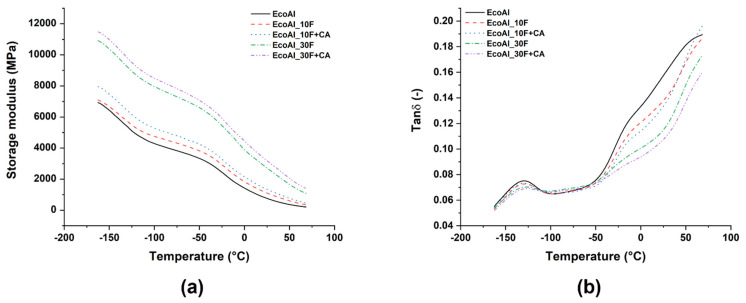
(**a**) Storage modulus and (**b**) tanδ curves as a function of temperature for all EcoAllene-based composites.

An increase in the storage modulus over the whole temperature range with the content of flax fibres (Figure 9a and Table 5) confirms tensile and bending tests’ outcomes, as a result of a stiffening of composites compared to unfilled EcoAllene due to a restricted chain mobility below and above the glass transition. To assess the effectiveness of flax fibres on the storage modulus of the composites, the coefficient *C* was calculated according to Equation (1) [48,49,50]:

(1) $$C=\frac{{\left(E_{g}^{\prime }/E_{r}^{\prime }\right)}_{comp}}{{\left(E_{g}^{\prime }/E_{r}^{\prime }\right)}_{matrix}}$$

where $E_{g}^{\prime }$ and $E_{r}^{\prime }$ are the storage modulus values in the glassy and rubbery region, respectively. The corresponding values are summarized in Table 5. The higher the value of the constant C, the lower the effectiveness of the filler. The data suggest that modified formulations are more effective with respect to non-modified ones, especially at high flax fibre content.

The position and magnitude of tanδ are indicative of the glass transition temperature (T_g_) and the damping properties of the composites, respectively [51]. Glass transition values, evaluated as the peak of tanδ in Figure 9b, were not affected by the introduction of flax fibres, featuring a value around −128 °C. This suggests that changes in the mobility of small segments of polymer chains did not occur. On the contrary, tanδ peak magnitude, which is linked to the energy dissipation ability of composites, was reduced with increasing fibre content and the presence of a coupling agent. Such a drop is ascribed to an improved interfacial adhesion and reduced polymer chain mobility [52], which supports the results of quasi-static mechanical characterizations.

## 4. Conclusions

Post-consumer aseptic multilayer packaging consisting of LDPE, HDPE, aluminium and traces of PP, as evaluated by differential scanning calorimetry, was used for the first time as a matrix for composites reinforced with short flax fibres up to a weight fraction of 0.3. Results suggest an increasing trend in stiffness and strength with flax fibre amount, which was enhanced even further by the modification of the matrix with a maleated coupling agent. The flax fibre/matrix interfacial adhesion, though limited in non-compatibilized blends, allowed an increase not only in terms of stiffness, but also in strength over the weight fractions investigated in the present study. SEM-based morphological assessment highlighted a strong level of interfacial adhesion when a coupling agent was used, resulting in remarkable improvements by 92% and 138% in tensile and flexural strength, respectively. This occurred without sacrificing the Charpy impact strength, which was only marginally affected, with all composite formulations featuring values around 5 kJ/m^2^. Limited nucleation ability of flax fibres was recorded by DSC, while the thermal stability was decreased due to the presence of less-thermally stable constituents, such as hemicellulose and cellulose. The onset degradation temperature was in any case higher than 300 °C. DMA analysis confirmed the results of quasi-static mechanical tests, highlighting an increase in storage modulus over the neat matrix and a great effectiveness of flax fibres. The developed low-cost thermoplastic composites show potential in automotive interiors or in components for public transportation and in all those applications traditionally covered by wood plastic composites, such as outdoor decking applications. In addition, this strategy based on mechanical recycling, specifically aimed at recovering plastic materials to be re-integrated in the life cycle of plastic components, represents the second best valuable strategy of waste management before energy recovery and disposal according to the European Directive on waste management.

## Figures and Tables

**Table 1:** List of all EcoAllene-based composites.

Specimen ID	EcoAllene (wt.%)	Flax Fibres (wt.%)	CA (wt.%)
EcoAl_neat	100	-	-
EcoAl_10F	90	10	-
EcoAl_10F + CA	85	10	5
EcoAl_30F	70	30	-
EcoAl_30F + CA	65	30	5

**Table 2:** T_5%_, T_10%_, and T_max_ for EcoAllene-based formulations obtained from TGA analysis.

Specimen ID	T_5%_ (°C)	T_10%_ (°C)	T_max_ (°C)
EcoAl_neat	438.1 ± 0.6	452.5 ± 1.1	484.6 ± 0.8
EcoAl_10F	319.5 ± 0.8	379.1 ± 0.7	482.3 ± 0.9
EcoAl_10F + CA	327.3 ± 0.7	398.5 ± 0.6	481.4 ± 0.7
EcoAl_30F	306.7 ± 0.8	326.1 ± 0.6	482.1 ± 0.7
EcoAl_30F + CA	310.8 ± 0.5	326.6 ± 0.7	483.2 ± 0.8

**Table 3:** Thermal properties of composites obtained from differential scanning calorimetry (DSC) analysis (T_m_ = melting temperature; T_c_ = crystallization temperature. Numbers 1 to 3 refer to the temperatures labeled in Figure 2).

Specimen ID	T_m1_ (°C)	T_m2_ (°C)	T_m3_ (°C)	T_c1_ (°C)	T_c2_ (°C)	T_c3_ (°C)
EcoAl_neat	108.6 ± 0.2	128.0 ± 0.1	163.9 ± 0.1	112.8 ± 0.1	92.7 ± 0.4	76.6 ± 0.3
EcoAl_10F	107.6 ± 0.1	128.4 ± 0.5	163.8 ± 0.1	113.4 ± 0.5	93.5 ± 0.1	76.5 ± 0.1
EcoAl_10F + CA	109.3 ± 0.9	129.4 ± 1.3	164.4 ± 0.6	112.4 ± 0.8	93.3 ± 1.1	75.8 ± 0.6
EcoAl_30F	108.9 ± 0.1	129.0 ± 0.1	164.3 ± 0.1	112.1 ± 0.1	92.1 ± 0.1	75.0 ± 0.3
EcoAl_30F + CA	109.9 ± 0.3	130.4 ± 0.4	165.0 ± 0.1	117.7 ± 0.1	92.4 ± 0.1	75.2 ± 0.2

**Table 4:** Summary of mechanical properties of polymer matrix composites reinforced with lignocellulosic fibres.

Matrix Type	Fiber Type	Coupling Agent	Tensile Modulus (GPa)	Tensile Strength (MPa)	Flexural Strength (MPa)	Flexural Modulus (GPa)	Fiber Content (wt.%)	Reference
HDPE	Hemp	yes	3.8	36.0	-	-	30	[40]
HDPE	Flax	no	4.6	21.6	43.6	5.1	40	[41]
HDPE	Moso bamboo	yes	4.7	29.5	52.5	5.3	60	[42]
HDPE	Wheat straw	yes	4.5	26.3	44.5	4.8	60	[42]
HDPE	Rice straw	yes	3.4	22.5	45.0	4.3	60	[42]
HDPE	Rice husk	yes	3.0	21.0	39.2	3.2	60	[42]
HDPE	Sugarcane bagasse	yes	3.4	23.5	48.0	4.1	60	[42]
HDPE	Cotton stalk	yes	3.6	26.5	48.1	4.3	60	[42]
HDPE	Wood flour	yes	2.5	18.6	37.1	2.7	65	[43]
HDPE	Hemp pomace	no	1.4	17.6	-	-	30	[44]
HDPE	Hemp pomace	yes	1.7	21.6	-	-	30	[44]
HDPE	Hemp straw	no	1.8	18.7	-	-	30	[44]
HDPE	Hemp straw	yes	2.2	24.7	-	-	30	[44]
HDPE	Linseed cake	no	0.7	17.5	-	-	20	[45]
PHBV	Hemp	no	3.8	37.6	-	-	15	[46]
PHBV	Hemp	yes	3.9	38.1	-	-	15	[46]
PHBV	Flax	no	3.5	36.3	-	-	15	[46]
PHBV	Flax	yes	3.9	36.9	-	-	15	[46]
PP	Abaca	yes	4.9	42.0	-	-	30	[47]
PP	Jute	yes	5.8	47.9	-	-	30	[47]
PHBV/Ecoflex	Abaca	no	4.4	28.0	-	-	30	[47]
PHBV/Ecoflex	Jute	no	7.0	35.2	-	-	30	[47]

HDPE: high-density polyethylene; PHBV: poly (3-hydroxybutyric-co-3-hydroxyvaleric acid); PP: polypropylene.

**Table 5:** Storage modulus and fibre effectiveness coefficients (C) of EcoAllene-based composites calculated according to Equation (1).

Specimen ID	$E_{g}^{\prime }$ (MPa) at −150 °C	$E_{g}^{\prime }$ (MPa) at 30 °C	C
EcoAl_neat	6420.75	658.99	-
EcoAl_10F	6647.76	988.39	0.69
EcoAl_10F + CA	7448.36	1207.78	0.63
EcoAl_30F	10,353.66	2481.93	0.43
EcoAl_30F + CA	10,990.59	2981.77	0.38

## Data Availability

The data are contained within this article.
